# Situs Inversus Totalis in the Critical Care Unit: A Case Report and Literature Review

**DOI:** 10.7759/cureus.45381

**Published:** 2023-09-16

**Authors:** Wilfredo M Pedreira-Garcia, Vanessa Vando-Rivera, Maria Rodriguez-Martinez, Andres Velazquez, Charlynne De Jesus Ramos, Yomayra Otero-Dominguez, William Rodriguez-Cintron, Francisco Del Olmo-Arroyo

**Affiliations:** 1 Internal Medicine, VA Caribbean Healthcare System, San Juan, PRI; 2 Pneumology and Critical Care Medicine, VA Caribbean Healthcare Systems, San Juan, PRI; 3 Internal Medicine, VA Caribbean Healthcare Systems, San Juan, PRI; 4 Critical Care Medicine, VA Caribbean Healthcare Systems, San Juan, PRI; 5 Infectious Disease, VA Caribbean Healthcare Systems, San Juan, PRI; 6 Pulmonary and Critical Care Medicine, VA Caribbean Healthcare System, San Juan, PRI; 7 Pulmonary and Critical Care Medicine, VA Caribbean Healthcare Systems, San Juan, PRI

**Keywords:** anatomic imaging, s: embryology, pneumology, critical care and hospital medicine, bronchoscopy, situs-inversus-totalis

## Abstract

Situs inversus is a rare congenital disorder where the reversal of some of the major thoracic or abdominal organs is present. In this disorder, alterations in the fetus's organ lateralization lead to a complete reversal in the arrangement of the internal organs. Most of the time, they are found incidentally when having a procedure or imaging modality. Little has been written regarding the challenges encountered while providing critical care to these patients. Here we present the case of a 68-year-old male patient admitted to the intensive care unit (ICU) with hypoxemic respiratory failure secondary to pneumonia who underwent diagnostic bronchoscopy for organism identification and was confirmed to have situs inversus totalis. Situs inversus totalis represents a challenge at different levels of care to these patients, including in the ICU. Limitations in critical care can be seen upon imaging identification, and during routine procedures performed at the ICU. Confusion might appear while performing bedside point of care ultrasound, obtaining vascular access, performing electrocardiogram, and sample identification, among others. The case brings the relevance of being able to recognize this rare disorder, which can be diagnosed even in advanced age since it might present the clinician with challenges at the time of providing care to patients.

## Introduction

Situs inversus is a rare congenital disorder where the transposition of some of the major thoracic or abdominal organs is present. Situs inversus totalis (SIT) is even more infrequent where there is a complete reversal in the arrangement of the internal organs [1]. It has an incidence of 1 in 8,000 births with a prevalence of 0.01% of the population. Even the most experienced and active surgeons can see between 1 to 2 cases at most in their lifetime [2]. They are usually found incidentally when having a procedure or imaging modality. Laterality is established early in development at the formation of the primitive streak during gastrulation. This process is dependent on a cascade of genes and signaling molecules. Although the definitive cause of situs inversus is unknown, multiple gene mutations have been associated with the condition including mutations in nodal and PITX2 genes, and it has also been involved with other disorders such as primary ciliary dyskinesia [1,3]. 

Any failure during this early development may lead to an array of different disorders varying from partial to complete inversion. Situs solitus refers to the normal position of abdominal and thoracic organs. Situs inversus totalis refers to the complete reversal or mirror imaging of the normal anatomical position of abdominal and thoracic organs. Situs ambiguous describes any other abnormal arrangement of abdominal and thoracic organs across the left-right axis [1]. This condition may present difficulties during diagnostic or therapeutic procedures and interventions. We herein present a case of a patient diagnosed with situs inversus totalis at an advanced age confirmed by imaging modalities and bronchoscopy whose care at the intensive care unit required modifications to current standardized interventions.

## Case presentation

**Figure 1:**
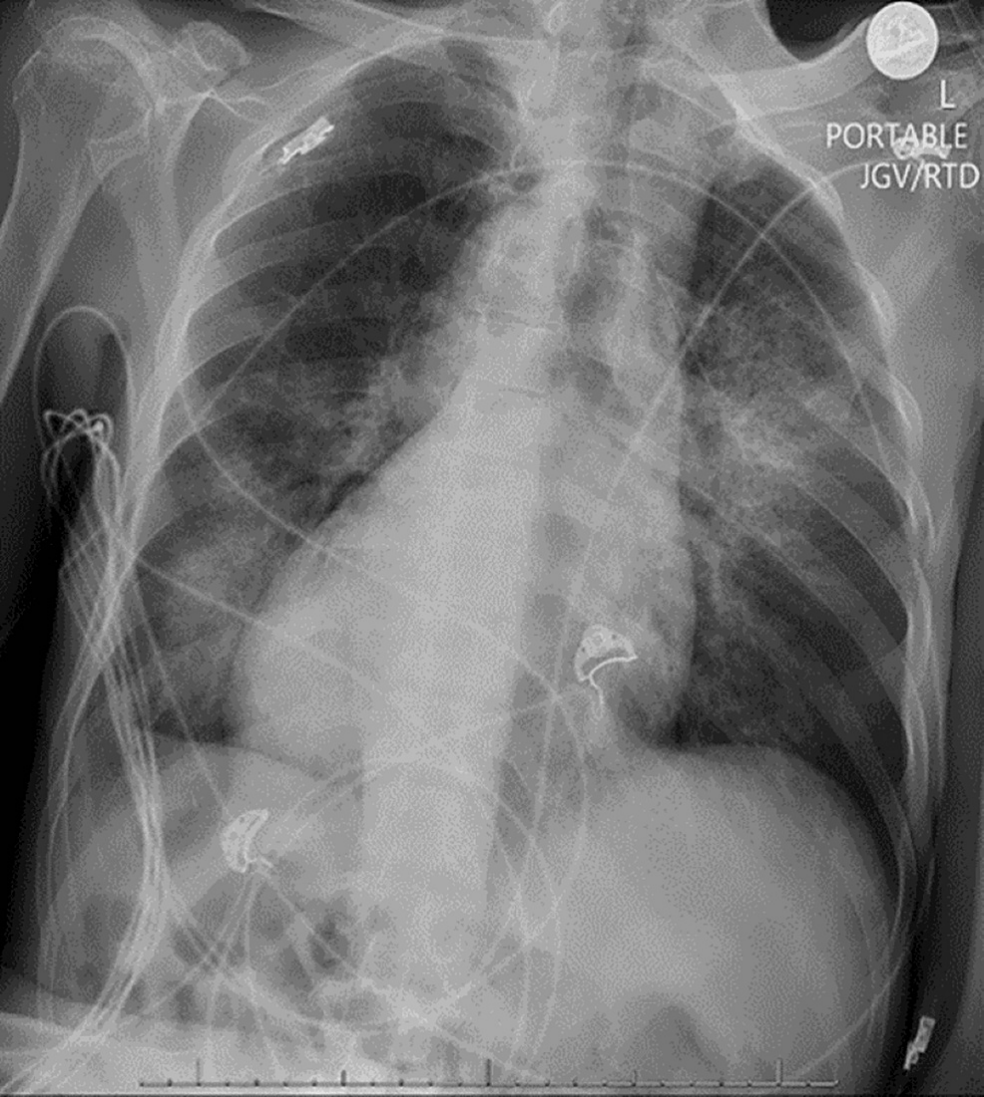
CI with opacities at right base & prominent cardiac silhouette with dextrocardia.

**Figure 2:**
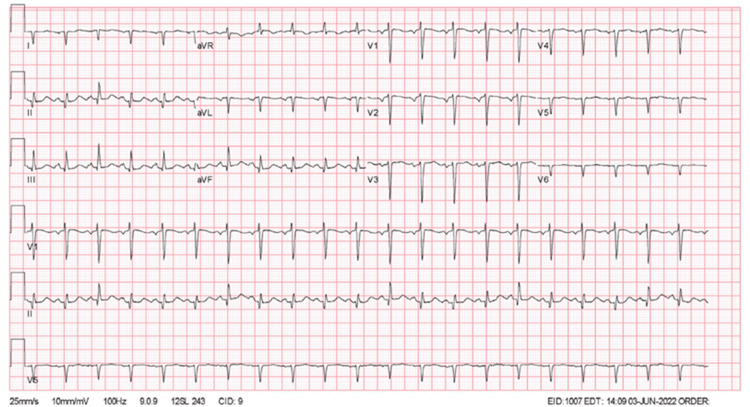
Initial electrocardiogram of patient. Noted positive aVR, and aVF, with negative I.

We present a case of a 68-year-old male with a previous history of dextrocardia, schizophrenia, and seizure disorder, noncompliant with medications who was brought to the emergency department after an episode of involuntary bilateral extremity jerky movements that lasted half an hour and then remained unconscious. The patient was endotracheally intubated and started on mechanical ventilation due to neurological status. Vital signs demonstrated a fever of 103.7°F, blood pressure (BP) 135/87 mmHg, heart rate (HR) 126 bpm, respiratory rate (RR) 28, O2 91%. Physical examination was most remarkable for Glasgow coma scale (GCS) of four (E1V1M2), an apical impulse at the right fifth intercostal space, and diffuse rhonchi more prominent on the right side. Laboratories were remarkable for leukocytosis of 12.6 with left shifting. Initial chest imaging (CI) (Figure 1) showed multiple multilobular consolidations, bilateral pleural effusions, and dextrocardia. An electrocardiogram (ECG) (Figure 2) was performed with leads on the left side and was read as sinus tachycardia, without acute ischemic changes. The patient was endotracheally intubated and started on mechanical ventilation due to neurological status. He was started on antiseizure medications and antibiotics.

**Figure 3:**
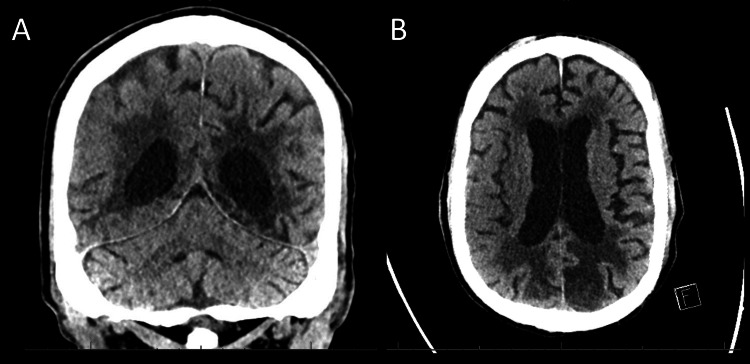
Head CT without contrast. Noted left and right chronic infarcts. (A) Demonstrate coronal view. (B) Demonstrate axial view.

**Figure 4:**
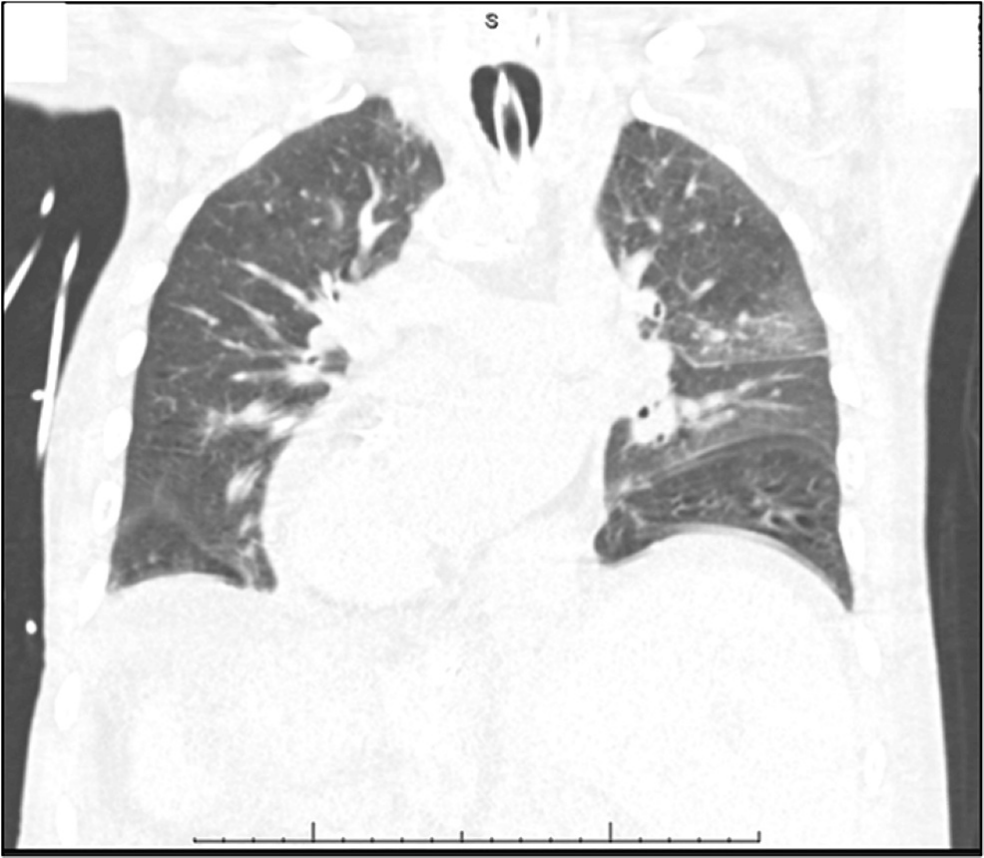
Chest CT demonstrating inverted anatomy.

**Figure 5:**
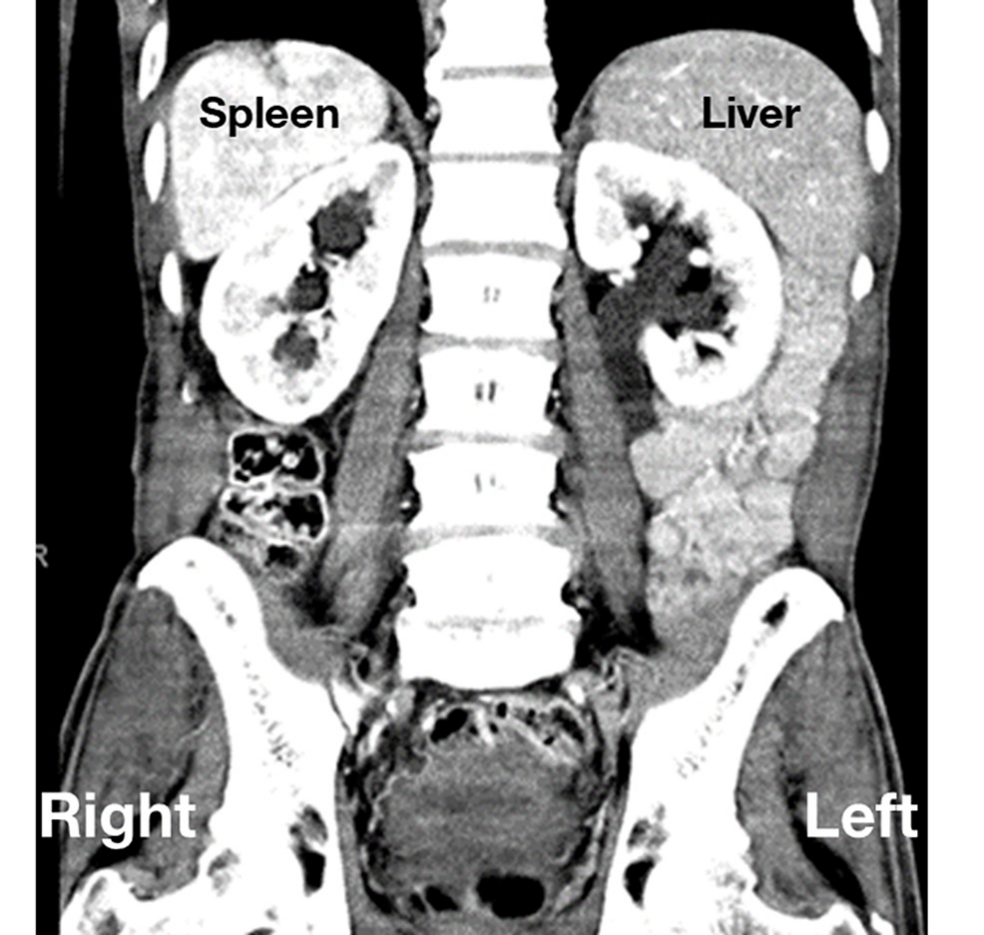
Abdominal imaging with noted right-sided spleen and left-sided liver.

He was admitted to the intensive care unit (ICU) for a higher level of care with a diagnosis of hypoxemic respiratory failure on mechanical ventilation with aspiration pneumonia, and post-status epilepticus. A head computerized tomography (CT) scan (Figure 3) was performed and did not find acute findings or differences in structures. The electroencephalogram (EEG) shows no signs of seizure. Blood, sputum, and urine cultures were persistently negative. Despite multiple days of antibiotics, the patient remained on mechanical ventilation without significant improvement. Chest CT (Figure 4) discrepancies in anatomy; right lung with an upper and lower lobe, the left lung with an upper, middle, and lower lobe, and known dextrocardia. This imaging was also remarkable for a left-sided liver and a right-sided spleen. Old Abdominal/pelvic CT (Figure 5) confirmed such findings. Given the complete inversion of both thoracic and abdominal organs, the patient at this time was identified with situs inversus totalis.

**Figure 6:**
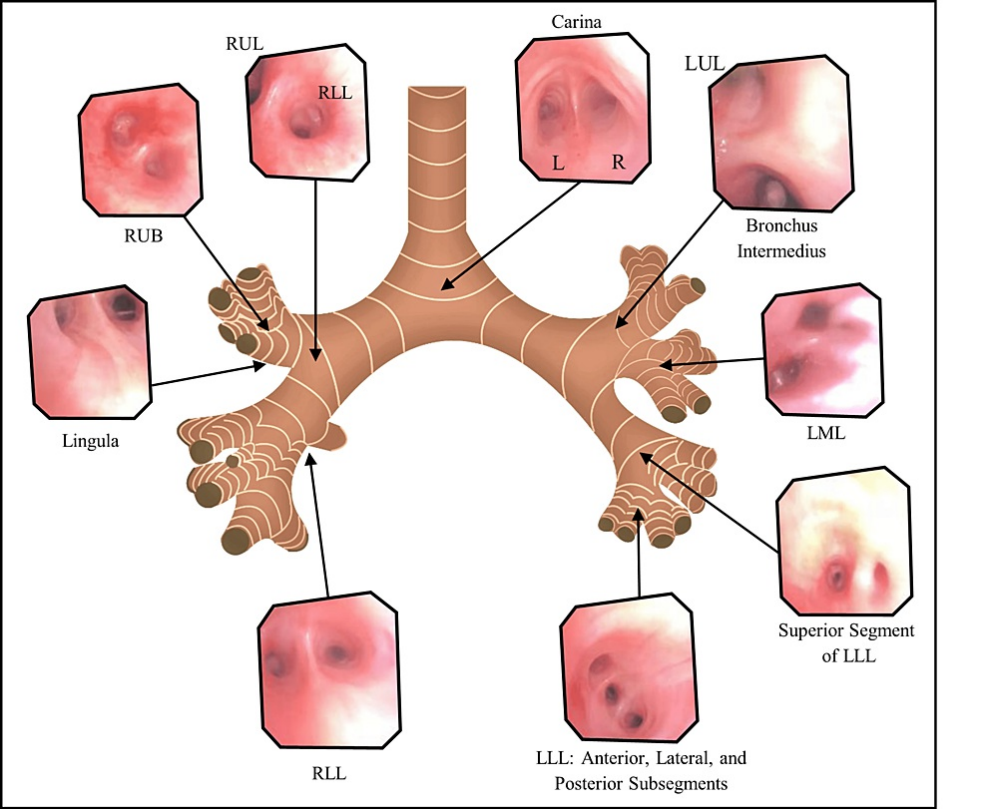
Bronchoscopy results obtain in our patient with SIT. L = Left; R = Right; LML = Left Middle Lobe; LLL = Left Lower Lobe; LUL = Left Upper Lobe; RLL = Right Lower Lobe; RUL = Right Upper Lobe.

Despite interventions, persistently negative cultures, and relatively unchanged imaging findings, bronchoscopy was performed (Figure 6) for organism identification. It was noted upon insertion of scope that the patient had a complete inversion of his bronchial tree, consistent with imaging findings. Segments were noted to be anatomical mirror images when compared to the general population. The left main bronchus was noted to be shorter, wider, and more vertical compared to the contralateral side. Bronchial lavages were performed after recognizing anatomical landmarks. However, they were negative for bacterial, atypical, or fungal organisms. He was treated with 10 days of antibiotics and diuretics to decrease pleural effusions with an improvement of symptoms and radiological findings; the patient was afterward successfully extubated. Despite interventions and stabilization, the patient's neurological status never recovered.

## Discussion

**Figure 7:**
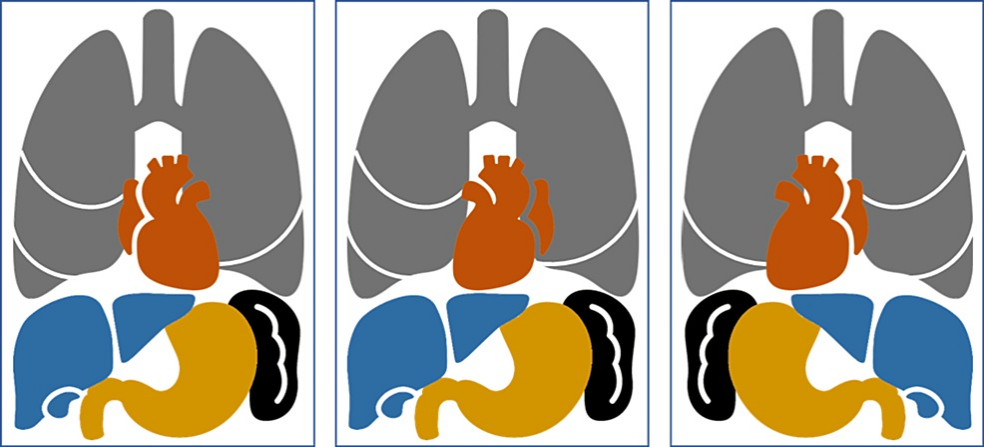
Anatomical variations. “A” demonstrates situs solitus which is the normal position of abdominal and thoracic organs. “B” demonstrates situs ambiguous describes any abnormal arrangement of abdominal and thoracic organs across the left-right axis. In this case, the heart is the only organ with a change in its axis. Lastly, “C” demonstrates situs inversus totalis, which is the complete transposition or mirror imaging of the normal anatomical position of abdominal and thoracic organs.

Situs inversus totalis (Figure 7) is a congenital condition that can be diagnosed even at an advanced age as seen in this patient. Family members were only aware of the patient’s dextrocardia. Despite being simply a transposition of internal organs, multiple questions and challenges arose while he was admitted to the critical care unit. 

When approaching the topic by organ systems, it can be noticed that the definition of SITs consists of the transposition of internal thoracic and abdominal organs, but not much information exists on other organs including the brain structure. Asymmetry of brain structure has not been identified. Interest in anomalies in imaging or EEG results has surged in recent years. In this patient with epilepsy due to multiple old ischemic infarcts, head CT grossly did not elucidate any anatomical variations or differences in structures. Nonetheless, EEG consists of placing electrodes on specific places of the scalp to determine cortical electrical activity in the specific place the lead is placed. This patient had increased metabolic activity in his EEG, but they were non-specific findings as it is a technique where cortical brain activity is read-only in a particular region [4]. Even a change in leads will not change the result as each region is identified by location in the scalp.

As per Vingerhoets et al., these claims are sustained as the brain tends to be symmetric and no differences can be grossly seen from an anatomical standpoint [5]. However, few studies have focused on brain asymmetries in normal and SIT anatomical variants. As per Geschwind et al., asymmetry of the brain's gray matter concentration can be seen in the dominant hemisphere. In normal anatomical variants, it is mentioned that they tend to present with increased gray matter concentration in the right frontal petalia, right planum temporale, and left occipital petalia than the respective contralateral side [6]. However, the asymmetry in these regions has not been consistently seen in individuals with SITs and data is limited. Hence gross anatomy by imaging alone cannot be used in SIT as it does not evaluate the functionality of the brain [7]. Furthermore, limited studies have been performed regarding the functionality of the brain in SIT individuals. Ihara et al. demonstrated that from a functional perspective using magnetoencephalography the pattern of language dominance in SIT in a study of three individuals comparing them to 11 subjects with normal anatomical variants differs [7]. Yet more studies regarding a link between human visceral and brain functional laterality are still needed as results are limited and some results demonstrate no significant link between them [5].

**Figure 8:**
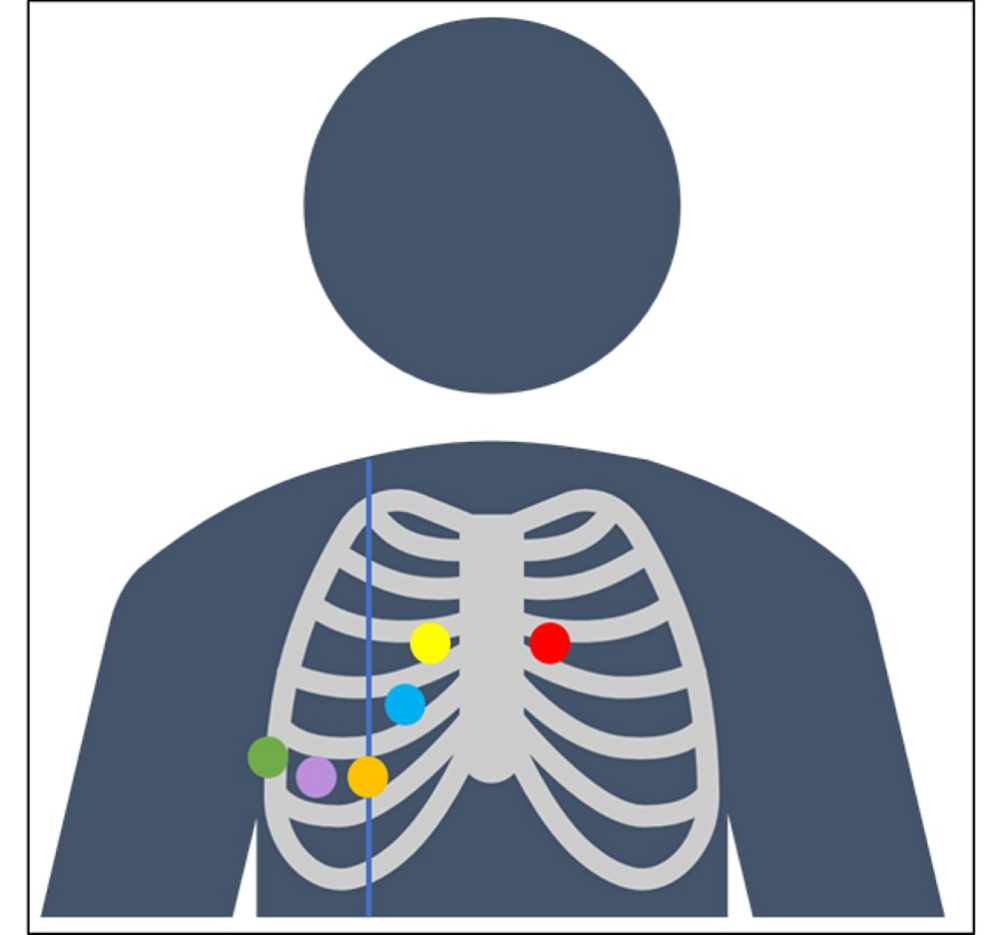
Adequate position of leads in a patient with SIT. By color: Red demonstrate lead V1, yellow lead V2, light blue lead V3, orange lead V4, purple lead V5, and green lead V6.

Contrary to the brain, dextrocardia in an individual can be easily diagnosed with proper physical and imaging findings as seen in this patient. However, despite knowing the transposition of the heart in this patient, ECG was initially performed on the patient’s left side as performed in the normal anatomical variant. However, information gained from this ECG initially in this unconscious patient where ischemic infarct was to be ruled out was limited as it only demonstrated the patient’s right ventricular conduction pattern as normally seen in a right-sided ECG. The correct orientation of leads can be seen in Figure 8 to obtain complete information about the heart conduction instead of a limited heart view. 

Although not seen in our patient, lethal arrhythmias or asystole are possible complications that patients can develop in the ICU. From a cardiovascular perspective, current cardiopulmonary resuscitation (CPR) guidelines encourage early and high-quality compressions with minimum interruption, but they do not mention deviations in treatment in cases with SIT or dextrocardia. One case report [8] postulated how a hypoxemic cardiac arrest in an individual with undiagnosed SIT required five cycles of CPR and shock in the apex-sternal (left anterolateral) position without return of spontaneous circulation. Using a point of care ultrasound (POCUS), the left parasternal short axis, long axis, and subcostal regions could not demonstrate the heart. It was only apparent on the subxiphoid view and the axis was towards the right side of the chest. With this identification, compressions and defibrillation were started to be given on the right side of the chest with the return of spontaneous circulation. Hence, the correct anatomical position is needed for adequate compressions. If POCUS is available, it may be used rapidly to exclude anatomical causes in a situation of proper CPR with ineffective outcomes.

**Figure 9:**
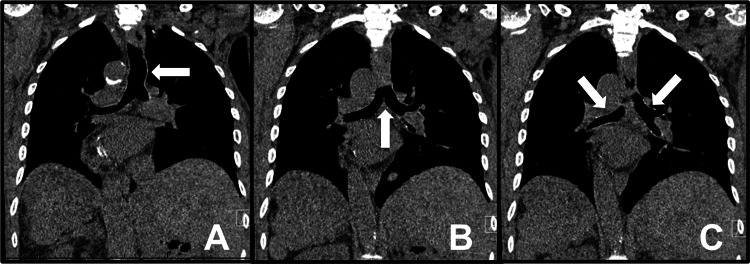
Appreciation of bronchial tree angulation. Ventral to dorsal CT images demonstrating by arrows the Trachea (A), Carina (B), and Right and Left primary bronchi (C). Left Primary Bronchi can be appreciated to have a more downward slope compared to the horizontal presentation seen on the Right Primary Bronchi in SIT.

Regarding the lung anatomy, the right main bronchus tends to be wider, shorter, and more vertical than its contralateral side, predisposing individuals to aspiration pneumonia or foreign body aspiration in the right lower lobe [9]. In individuals with SIT, by anatomy, pulmonary infiltrates in the left side can be suggestive of aspiration pneumonia. Additionally, information regarding surgical lung interventions and bronchoscopy in individuals by SIT is limited. Bronchoscopies can be performed to confirm the anatomic variant of the tracheobronchial tree before performing surgical intervention, such as decortication of pleural or parenchymal masses [10-11]. Additionally, no specific guidelines exist at the moment for reporting findings. In our patient, following the bronchus on CT imaging (Figure 9) confirmed that the left main bronchus was shorter, wider, and more vertical than the right main bronchus. With regards to the bronchoscopy, it was confirmed upon insertion of scope that the patient had a complete inversion of his bronchial tree as previously seen in Figure 6. Segments were anatomical mirror images and recognizing anatomical landmarks was important before passing scope through the lung. Furthermore, laboratory labeling of samples was questioned by hospital staff given confusing anatomy such as “right lingula”, and “left middle lobe”, creating confusion and doubt while reporting the information. For this reason, we suggest that labeling and documentation must include “situs inversus variant” to minimize errors in documentation.

In the abdominal/pelvic anatomy, our patient did not exhibit abdominal or pelvic discomfort, but previous Abd/Pelvic CT imaging (Figure 5) reflected intra-abdominal transpositions of visceral organs. In the critical care setting, it must be noted that when pain is present it will reflect on the organ’s ipsilateral side. For example, pain in the left upper quadrant can be acute cholecystitis, and pain in the left lower quadrant can be appendicitis instead of diverticulitis as reported in numerous cases [12-14]. In this manner, ultrasound usage can aid in orientation. Surgeons approach individuals with visceral transposition with difficulty. These difficulties include changing port placement when performing laparoscopic cholecystectomy, as well as changing dissection planes that are normally performed with a right-handed hand that now require to be performed by the left hand and may even require more than one assistant [15]. 

Lastly, there have been few case experiences, as seen above, regarding changes in techniques or surgery strategies for individuals with SIT. Furthermore, there is a lack of literature on patient safety in SIT or situs ambiguous. We herein suggest that for each patient diagnosed with transposition of organs, a medical flag must be placed on the patient’s record to avoid subsequent confusion and to provide the best possible care for each patient. For example, for a patient presenting to the emergency department, EKG leads could be placed correctly on the first try without delay. In addition, if a patient presents with pain in the abdominal region, the differential diagnosis changes knowing there is a transposition of vessels. Last of all if a procedure must be performed, meticulous planning is needed, and samples must be correctly identified. If there is a prior notification in the system notifying the patient’s anatomic variance, samples can be correctly labeled and received without confusion as seen in our patient.

## Conclusions

When evaluating an individual with situs inversus totalis confusion can arise as the anatomy is a mirror image. Clinical history and findings in the examination as well as imaging are important for any clinicians in the management of any patients, specifically with anatomical variants present. POCUS is highly encouraged to determine the correct orientation. There are few documented cases in the literature that do not show a high incidence of critical care illnesses or information regarding patient safety. For this reason, our case raises awareness of this rare congenital disorder that can be diagnosed even in advanced age as an important anatomical variant that many would not see in a lifetime and the need to establish the right work plan in the ICU setting, where precision, accuracy and time is the utmost importance.
